# The association between metabolic components and markers of inflammatory and endothelial dysfunction in adolescents, based on the Ewha Birth and Growth Cohort Study

**DOI:** 10.1371/journal.pone.0233469

**Published:** 2020-05-20

**Authors:** Hye Ah Lee, Eun Jeong Choi, Bohyun Park, Hwayoung Lee, Young Sun Hong, Hae Soon Kim, Moon-Kyung Shin, Hyesook Park

**Affiliations:** 1 Clinical Trial Center, Mokdong Hospital, Ewha Womans University, Seoul, Korea; 2 Department of Preventive Medicine, College of Medicine, Ewha Womans University, Seoul, Korea; 3 Department of Anatomy, College of Medicine, Ewha Womans University, Seoul, Korea; 4 Department of Internal Medicine, College of Medicine, Ewha Womans University, Seoul, Korea; 5 Department of Pediatrics, College of Medicine, Ewha Womans University, Seoul, Korea; 6 Department of Food and Nutrition, Kyung Hee University, Seoul, South Korea; University of Luebeck, GERMANY

## Abstract

We assessed the association between metabolic health and markers of inflammation and of endothelial dysfunction using data from the Ewha Birth and Growth Cohort Study. The data of 195 subjects aged 13–15 years were analyzed. To assess metabolic syndrome, continuous metabolic syndrome (cMets) scores were calculated. We measured the levels of high-sensitivity C-reactive protein (hs-CRP), intercellular adhesion molecule 1 (ICAM-1), and vascular cell adhesion molecule 1 (VCAM-1) as markers of inflammation and endothelial dysfunction. An increase of one SD in the cMets score resulted in a 1.25-fold (95% CI 1.10–1.42) increase in the risk of acute inflammatory status and a 1.26-fold (95% CI 1.11–1.43) increase in the risk of endothelial dysfunction as defined by ICAM-1, while VCAM-1 showed a meaningless trend. Of the metabolic components, body mass index (BMI) was positively associated with elevated hs-CRP levels and high-density lipoprotein cholesterol (HDL-c) levels were negatively associated with elevated ICAM-1 levels. Additionally, a mediation analysis showed that a high BMI was directly related to elevated hs-CRP levels and indirectly related to elevated ICAM-1 levels via HDL-c. Our findings show that poor metabolic health was related to an unfavorable inflammatory status and endothelial dysfunction in adolescents.

## Introduction

Metabolic syndrome is a clustering of obesity, high blood pressure, poor glucose tolerance, and dyslipidemia, and is a risk factor for cardiovascular disease (CVD). Longitudinal studies have suggested that metabolic health in later life can be traced back to childhood [1–3]. However, it is difficult to assess disease susceptibility in growing children, who vary physically and metabolically. Moreover, the prevalence of metabolic syndrome is < 5% in children and adolescents [4] and so a study requires a large sample size. The continuous metabolic syndrome (cMets) score has been proposed as an index of metabolic syndrome in children or adolescents [5] and has been evaluated extensively [2,6,7].

Regarding the effect of metabolic health on CVD development, abnormal metabolic components contribute to endothelial dysfunction accompanied by inflammation of the vessel wall, leading to the development of atherosclerosis and CVD [8]. Based on these assumptions, the associations between metabolic components and inflammatory markers (*e*.*g*., high-sensitivity C-reactive protein [hs-CRP], interleukin-6 [IL]-6) [6,9–11], and markers of endothelial dysfunction (*e*.*g*., intercellular adhesion molecule [ICAM-1] and vascular cell adhesion molecule [VCAM-1]) [6,11,12] have been evaluated. However, most studies examined adults [10,11] or had case–control designs [12,13]. Although a previous study evaluated the relationships among hs-CRP, ICAM-1, VCAM-1, and cMets in adolescents [6], evidence for children or adolescents in general is lacking.

Therefore, to determine whether a poor metabolic status is linked to levels of preclinical markers of CVD in adolescents, we assessed the cross-sectional association between metabolic components and markers of inflammation and endothelial dysfunction using data from the Ewha Birth and Growth Cohort Study. Furthermore, to identify an easily implementable and effective intervention strategy, we used mediation analysis to evaluate the direct/indirect association between BMI and an unfavorable inflammatory status and endothelial dysfunction.

## Materials and methods

### Subjects

This study was part of the Ewha Birth and Growth Cohort Study. This cohort was established in 2001–2006 (baseline n = 940) at the Ewha Woman’s University Mokdong Hospital (Seoul, South Korea) and was designed to investigate risk and preventive factors related to growth and disease susceptibility. Detailed information on the cohort composition and methodology has been provided elsewhere [14,15]. In 2015, a follow-up program with subjects 13 years of age was begun that involved anthropometric measurements, questionnaires, dietary surveys, and blood and urine sampling. Blood sampling was conducted after an overnight fast. During the follow-up at 13–15 years of age, blood levels of hs-CRP, ICAM-1, and VCAM-1 were measured as preclinical markers of CVD. From 2015 to 2019, 217 subjects aged 13–15 years participated in follow-up; all and their parents or guardians provided written informed consent for participation. All data was completely anonymous before author access and analysis.

For this study, subjects who met any of the following criteria were excluded: missing data for hs-CRP, ICAM-1, and VCAM-1 (n = 22), or missing data for metabolic components (n = 8). Accordingly, complete data were available for 195 subjects (93 boys and 102 girls; 175, 16, and 4 aged 13, 14, and 15 years, respectively). The study protocol was approved by the Institutional Review Board of Ewha Womans University Hospital (no. SEUMC 2019-04-034).

### Indicators of metabolic health

The cMets scores were calculated based on body mass index (BMI); levels of high-density lipoprotein cholesterol (HDL-c), triglyceride (TG), and fasting glucose; and the mean arterial blood pressure (MAP). BMI (kg/m^2^) was calculated from the height and weight measured with the subjects wearing no shoes and light clothing using a stadiometer and a calibrated scale (GL-150, *G-Tech* International Co., Ltd., Uijeongbu, South Korea). The HDL-c, TG, and fasting glucose levels were measured using an automatic analyzer (Olympus AU2700; Beckman Coulter Inc., Fullerton, CA, USA). Blood pressure (BP) was measured by a trained nurse using an automatic device (BPBIO320, InBody Co., Ltd., Seoul, South Korea) with the subject in a stable position. MAP was estimated using the following formula: [(systolic BP − diastolic BP) ÷ 3] + diastolic BP.

To calculate cMets scores, the standardized z-score for the mean and standard deviation of each of five metabolic components was calculated stratified for sex but not age because the number of 14- or 15-year-old subjects was small (n = 20). Because the HDL-c level is inversely related to the cMetS score, the standardized HDL-c level was multiplied by −1. To calculate individual cMets scores, the standardized z-scores for the five components were summed. A relatively high cMets score indicates an unfavorable metabolic syndrome status.

### Outcomes

We considered hs-CRP, ICAM-1, and VCAM-1 levels as the outcome variables. The hs-CRP level in fasting blood was measured by particle-enhanced immune turbidimetric assay (Cobas 8000 C702 analyzer, Roche, Mannheim, Germany). The limit of detection (LOD) for hs-CRP was 0.15 mg/dL, and values below the LOD were imputed using the equation: LOD / √2. The ICAM-1 (DCD540, R&D Systems, Minneapolis, MN, USA) and VCAM-1 (ECM340, Millipore, Billerica, MA, USA) levels were measured using commercial enzyme-linked immunosorbent assay (ELISA) kits. Measurements of hs-CRP, ICAM-1, and VCAM-1 levels were conducted at Seegene Medical Foundation (Seoul, South Korea). In terms of quality assurance/quality control, the coefficient of variance was < 10% for all measurements. The outcome variables were evaluated continuously. Additionally, we used alternative definitions of an unfavorable inflammatory status and endothelial dysfunction because of the lack of a clinical cutoff value for children and adolescents. Endothelial dysfunction was defined as a level of ICAM-1 or VCAM-1 ≥ 75th percentile. An acute inflammatory status was defined as an hs-CRP level of > 0.3 mg/dL [10,16], which is typically used for adults.

### Covariates

As demographic factors, sex, age, and monthly household income (< 3 million, 3–5 million, and ≥ 5 million KRW) were evaluated. Using a questionnaire, we collected data on the parental history of disease, physical activity, and food intake. Total energy intake for the past year was obtained from a semi-quantitative food-frequency questionnaire (FFQ) and consisted of 92 food items. The FFQ instrument was developed by expert consensus by adding beverage items (*e*.*g*., coffee intake) to the existing validated FFQ instrument [17,18]. Depending on the nature of the cohort study, the same investigative tools were used, with appropriate modifications for the age of the subjects. Individual dietary data were collected by trained dieticians during face-to-face interviews. Eating breakfast was related to a healthy dietary pattern and dietary quality [19,20], so eating breakfast daily was included as a covariate. The daily amount of time spent sedentary (*e*.*g*., watching TV, gaming) was divided into < 2 h and ≥ 2 h based on the distribution of data. The frequency of weekly vigorous-intensity physical activity (*e*.*g*., jogging, soccer, basketball, swimming) was categorized as never, 1–2 times/week, 3–4 times/week, and ≥ 5 times/week for > 20 min at a time. Exposure to secondhand smoke in households was investigated using the questionnaire.

### Statistical analysis

Summary statistics are presented as means ± standard deviation (SD) for normally distributed variables, medians with interquartile range (IQR) for non-normally distributed variables, and frequencies with percentage for categorical variables. Comparison of basic characteristics according to sex was performed by Student’s *t*-test, Mann–Whitney *U*-test, and chi-squared test.

To assess the linear associations of metabolic syndrome score and metabolic components with the outcome variables, we used the Pearson correlation for ICAM-1 and the Spearman correlation for hs-CRP and VCAM-1 based on the presence or absence of a normal distribution. The effects of basic characteristics on cMets scores were estimated as a coefficient with standard error (SE) by linear regression analysis.

The association of metabolic health with inflammation and endothelial dysfunction was assessed by multiple linear regression and multiple logistic regression. Because VCAM-1 levels were not normally distributed, the values were log-transformed. Of the subjects, 50.8% had an hs-CRP level lower than LOD, so it was evaluated by multiple logistic regression. We included sex, age at participation in follow-up, monthly household income, parental history of hypertension, parental history of diabetes, weekly vigorous-intensity physical activity, TV viewing, secondhand smoking, eating breakfast, and total energy intake as covariates. Multicollinearity was assessed by variance inflation factor (VIF), which was satisfied (VIF < 2 in all models).

Furthermore, we evaluated the mediating effects of biochemical metabolic factors on the association between BMI and an unfavorable inflammatory status and endothelial dysfunction. Log-transformed TG values were used in the analysis. The covariates included were as mentioned above. All statistical analyses were conducted using SAS software (ver. 9.4; SAS Institute, Cary, NC, USA). Statistical significance was determined as a value of *p* < 0.05 in a two-tailed test.

## Results

Of the 195 children, 47.7% (n = 93) were boys; the mean age of the subjects was 13.1 (± 0.4) years. Of the subjects, 23.1% and 14.4% had a parental history of hypertension and diabetes, respectively. Regarding weekly vigorous-intensity physical activity, 19.7% of the subjects responded “never” and 9.8% responded “> 5 times per week”. Of the subjects, 35.1% watched TV for more than 2 h a day, and 61.9% stated that they ate breakfast every day. Other than physical activity, there was no difference in basic characteristics according to sex (Table 1). Also, the basic characteristics were not associated with cMets scores (Table 2).

**Table 1:** Basic characteristics of the subjects.

	Total	Boys	Girls	*p*
	(n = 195)	(n = 93, 47.69%)	(n = 102, 52.31%)	
Age, years	13.12 ± 0.39	13.14 ± 0.41	13.11 ± 0.37	0.57
Menarche	NA	NA	88 (86.3%)	-
Monthly household income, KRW				
< 3 million KRW	12 (6.35%)	6 (6.52%)	6 (6.19%)	0.96
3–5 million KRW	56 (29.63%)	28 (30.43%)	28 (28.87%)	
≥ 5 million KRW	121 (64.02%)	58 (63.04%)	63 (64.95%)	
Maternal education level				
Graduated from high school	37 (19.68%)	17 (18.68%)	20 (20.62%)	0.88
Some college or higher	151 (80.32%)	74 (81.32%)	77 (79.38%)	
Parental disease history				
HTN	45 (23.08%)	17 (18.28%)	28 (27.45%)	0.18
DM	28 (14.36%)	13 (13.98%)	15 (14.71%)	> 0.99
Weekly vigorous-intensity physical activity				
Never	38 (19.69%)	12 (12.90%)	26 (26.00%)	0.02
1–2 times/week	73 (37.82%)	32 (34.41%)	41 (41.00%)	
3–4 times/week	63 (32.64%)	36 (38.71%)	27 (27.00%)	
≥ 5 times/week	19 (9.84%)	13 (13.98%)	6 (6.00%)	
TV viewing				
≥ 2 h/day	67 (35.08%)	30 (32.61%)	37 (37.37%)	0.59
Secondhand smoking				
Yes	17 (8.90%)	5 (5.43%)	12 (12.12%)	0.17
Total energy intake, kcal/d	2160.00 ± 594.22	2197.59 ± 523.46	2125.83 ± 652.72	0.40
Eating breakfast				
Everyday	117 (61.90%)	59 (65.56%)	58 (58.59%)	0.40

***Abbreviations*:** KRW, Korean Won; HTN, hypertension; DM, diabetes mellitus; NA, not applicable.

**Table 2:** Effect of subjects’ characteristics on cMetS scores.

	Coefficient (SE)	*p*
Age, years	0.27 (0.53)	0.60
Sex		
Boys	ref	
Girls	0.00 (0.41)	> 0.99
Monthly household income, KRW		
<3 million KRW	ref	
3-<5 million KRW	1.23 (0.91)	0.18
5≥ million KRW	0.74 (0.86)	0.40
Maternal education level		
Graduated from high school	ref	
Some college or higher	-0.52 (0.52)	0.32
Parental disease history, HTN		
No	ref	
Yes	0.11 (0.48)	0.82
Parental disease history, DM		
No	ref	
Yes	0.53 (0.58)	0.36
Weekly vigorous-intensity physical activity		
never	ref	
1–2 times/week	0.54 (0.57)	0.34
3–4 times/week	-0.54 (0.58)	0.35
≥5 times/week	0.69 (0.79)	0.39
TV viewing		
< 2 hours/day	ref	
≥ 2 hours/day	-0.06 (0.43)	0.89
Secondhand smoking		
No	ref	
Yes	-0.13 (0.73)	0.85
Total energy intake, per 100kcal	0.03 (0.04)	0.47
Eating breakfast		
Others	ref	
Everyday	-0.34 (0.43)	0.42

***Abbreviation*:** S.E, standard error; KRW, Korean Won; HTN, hypertension; DM, diabetes mellitus.

Summary statistics of metabolic components and markers of inflammation and endothelial dysfunction, including cMets scores, are listed in Table 3. The mean cMets score was comparable in boys and girls. Among metabolic components, the average TG level was significantly higher in girls than in boys, but other components did not differ significantly. Of the subjects, 8.7% (n = 17) had a BMI of ≥ 25.0 kg/m^2^. Mean hs-CRP, ICAM-1, and VCAM-1 levels were 0.1 mg/dL, 203.9 ng/mL, and 878.2 ng/mL, respectively, and were higher in boys than in girls. The mean differences by sex were significant for hs-CRP (*p* = 0.03) and VCAM-1 (*p* < 0.001). cMets scores were positively correlated with hs-CRP and ICAM-1 levels but negatively correlated with VCAM-1 levels. In addition, the correlation between BMI and hs-CRP was moderate (*r*_s_ = 0.43, *p* < 0.001) and that between HDL-c and hs-CRP (*r*_s_ = −0.20, *p* < 0.01) and ICAM-1 (*r* = −0.26, *p* < 0.01) was weak (Table 3).

**Table 3:** Descriptive statistics and correlation coefficients between metabolic syndrome components, scores, and markers of inflammation and endothelial dysfunction.

	Descriptive summary				Correlation coefficient		
	Total	Boys	Girls	*p*	hs-CRP^a^	ICAM-1	VCAM-1^a^
	(n = 195)	(n = 93, 47.69%)	(n = 102, 52.31%)				
Metabolic components							
BMI, kg/m^2^	20.39 ± 3.14	20.50 ± 3.37	20.29 ± 2.93	0.64	0.43 (*p*<0.001)	0.11 (*p* = 0.13)	-0.16 (*p* = 0.02)
MAP, mmHg	81.17 ± 8.47	81.83 ± 8.97	80.56 ± 7.98	0.30	0.09 (*p* = 0.21)	0.15 (*p* = 0.04)	0.09 (*p* = 0.23)
Fasting glucose, mg/dL	93.05 ± 6.53	93.19 ± 5.85	92.92 ± 7.12	0.77	-0.14 (*p* = 0.06)	0.11 (*p* = 0.11)	0.08 (*p* = 0.25)
TG, mg/dL	62.00	56.00	66.50	0.02	0.01 (*p* = 0.88)	0.08 (*p* = 0.28)	-0.16 (*p* = 0.02)
	(49.00–84.00)	(44.00–78.00)	(54.00–88.00)				
HDL-c, mg/dL	52.10 ± 9.77	52.25 ± 8.64	51.96 ± 10.73	0.84	-0.20 (*p*<0.01)	-0.26 (*p*<0.01)	0.02 (*p* = 0.84)
cMets score	0.00 ± 2.83	0.00 ± 2.85	0.00 ± 2.83	> 0.99	0.20 (*p*<0.01)	0.26 (*p*<0.01)	-0.06 (*p* = 0.45)
hs-CRP, mg/dL	0.11 (0.11–0.38)	0.19 (0.11–0.45)	0.11 (0.11–0.33)	0.03	-	-	-
ICAM-1, ng/mL	203.88 ± 65.24	211.51 ± 63.44	196.92 ± 66.38	0.12	-	-	-
VCAM-1, ng/mL	878.20	945.99	802.26	<0.001	-	-	-
	(760.53–1039.26)	(852.51–1109.51)	(716.39–939.63)				

***Abbreviations*:** BMI, body mass index; MAP, mean arterial pressure; TG, triglyceride; HDL-c, high-density lipoprotein cholesterol; hs-CRP, high-sensitivity C-reactive protein; ICAM-1, intercellular adhesion molecule; VCAM-1, vascular cell adhesion molecule; cMets, continuous metabolic syndrome.

^a^Correlation coefficients and *p*-values were obtained by the Spearman correlation method.

The associations between metabolic health and markers of endothelial dysfunction are listed in Table 4. The ICAM-1 level tended to increase by 5.6 (SE 1.6) as the cMets score increased by one SD, while the ICAM-1 level tended to decrease by 1.4 (SE 0.5) as the HDL-c level increased by 1 mg/dL. The metabolic components, including cMets scores, were not associated with log-transformed VCAM-1 levels.

**Table 4:** Results of multiple regression analysis of the effect of metabolic syndrome score and its components on the levels of markers of endothelial dysfunction in adolescents.

		Outcome variables	
	Independent variables	ICAM-1	VCAM-1 ^b^
Model 1^a^	cMets score	5.58 (1.64)**	0.0003 (0.01)
Model 2 ^a^	BMI, kg/m^2^	0.73 (1.71)	-0.01 (0.01)
	MAP, mmHg	0.41 (0.60)	0.002 (0.002)
	Fasting glucose, mg/dL	0.33 (0.74)	0.003 (0.003)
	TG, mg/dL	0.12 (0.14)	-0.0002 (0.001)
	HDL-c, mg/dL	-1.36 (0.53)*	-0.002 (0.002)

***Abbreviation*:** BMI, body mass index; MAP, mean arterial pressure; TG, triglyceride; HDL-c, high-density lipoprotein cholesterol; hs-CRP, high-sensitivity C-reactive protein; ICAM-1, intercellular adhesion molecule; VCAM-1, vascular cell adhesion molecule; cMets, continuous metabolic syndrome.

Results are coefficients with standard errors.

*p < 0.05,

**p < 0.01.

^a^Coefficients and *p*-values were obtained after adjustment for sex, age, monthly household income, parental history of hypertension, parental history of diabetes, weekly vigorous-intensity physical activity, TV viewing, secondhand smoking, eating breakfast, and total energy intake.

^b^VCAM-1 did not satisfy a normal distribution, and so was log transformed.

To consider inflammatory and endothelial dysfunction status, the effect of metabolic health was assessed as an odds ratio (OR) under controlling covariates (Table 5). As the cMets score increased by one SD, the risk of acute inflammatory status increased 1.25-fold (95% confidence interval [CI] 1.10–1.42) and the risk of endothelial dysfunction increased 1.26-fold (95% CI 1.11–1.43) based on ICAM-1. Among the metabolic components, BMI was positively associated with levels of markers of acute inflammation (OR = 1.35, 95% CI 1.17–1.56). In addition, the higher the HDL-c level, the greater the protective effect against endothelial dysfunction based on ICAM-1 (OR = 0.95, 95% CI 0.90–0.99). However, none of the metabolic markers, including cMets scores, was significantly associated with elevated VCAM-1 levels.

**Table 5:** Adjusted odds ratios for endothelial dysfunction and risk of acute inflammation according to the metabolic syndrome score and its components in adolescents.

		Outcome variables		
	Independent variables	hs-CRP^b^	ICAM-1^c^	VCAM-1^c^
Model 1^a^	cMets score	1.25 (1.10–1.42)**	1.26 (1.11–1.43)**	0.98 (0.86–1.11)
Model 2^a^	BMI, kg/m^2^	1.35 (1.17–1.56)**	1.11 (0.98–1.27)	0.88 (0.76–1.01)
	MAP, mmHg	1.04 (0.99–1.09)	1.01 (0.97–1.06)	1.03 (0.98–1.07)
	Fasting glucose, mg/dL	0.98 (0.93–1.04)	1.01 (0.96–1.07)	1.01 (0.94–1.07)
	TG, mg/dL	0.99 (0.98–1.00)	1.00 (0.99–1.01)	1.00 (0.99–1.01)
	HDL-c, mg/dL	0.97 (0.93–1.02)	0.95 (0.90–0.99)*	0.99 (0.95–1.04)

***Abbreviation*:** BMI, body mass index; MAP, mean arterial pressure; TG, triglyceride; HDL-c, high-density lipoprotein cholesterol; hs-CRP, high-sensitivity C-reactive protein; ICAM-1, intercellular adhesion molecule; VCAM-1, vascular cell adhesion molecule; cMets, continuous metabolic syndrome.

Results are odds ratios with 95% confidence intervals.

*p < 0.05,

**p < 0.001.

^a^Odds ratio and 95% confidence intervals were calculated after adjustment for sex, age, monthly household income, parental history of hypertension, parental history of diabetes, weekly vigorous-intensity physical activity, TV viewing, secondhand smoking, eating breakfast, and total energy intake.

^b^Acute inflammatory status was defined as a level > 0.3 mg/dL.

^c^Endothelial dysfunction was alternatively defined as ≥ 75th percentile.

**Fig 1:**
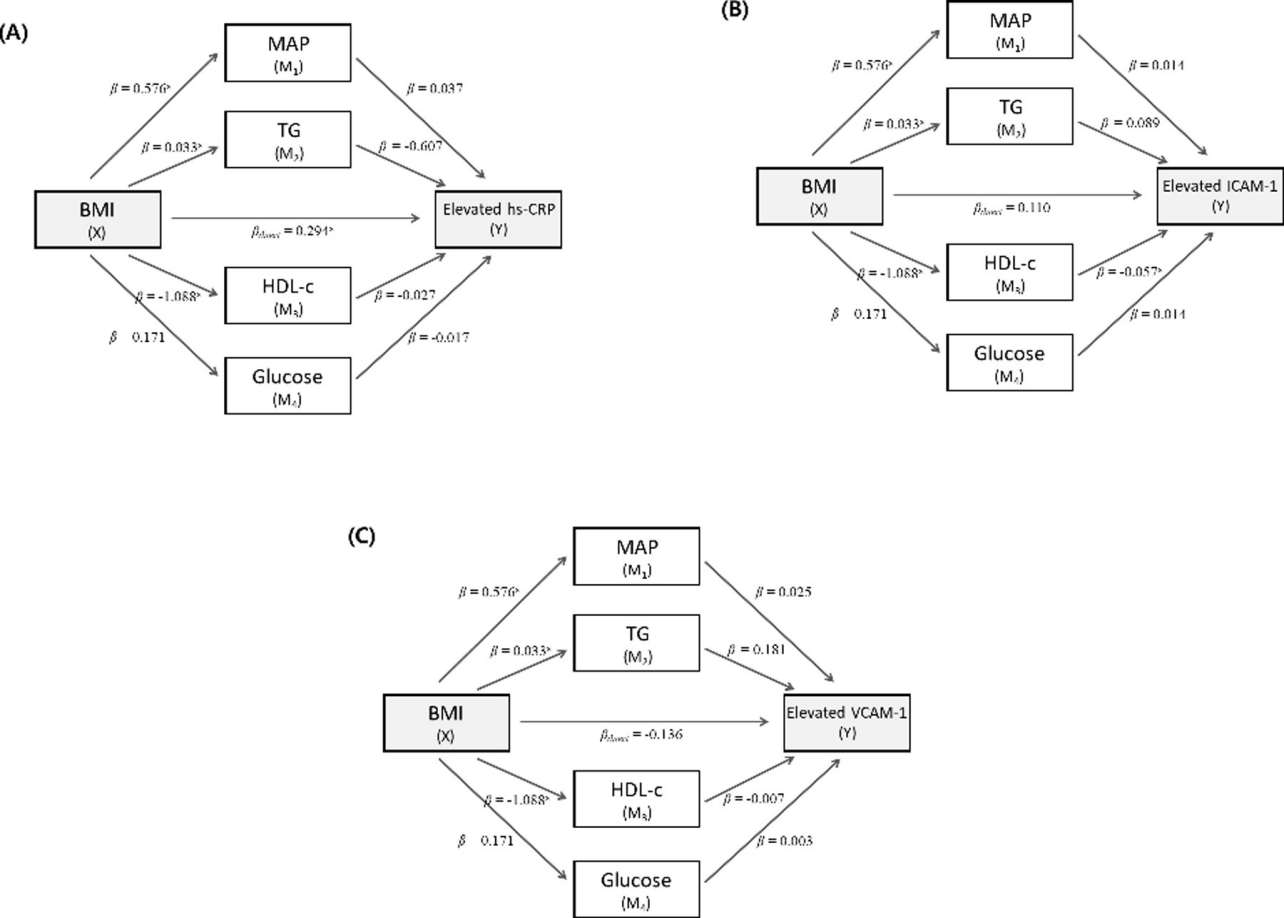
Effects of metabolic factors on the association between BMI and elevated levels of hs-CRP (A), ICAM-1 (B), and VCAM-1 (C). ***Abbreviation*:** BMI, body mass index; MAP, mean arterial pressure; TG, triglyceride; HDL-c, high-density lipoprotein cholesterol; hs-CRP, high-sensitivity C-reactive protein; ICAM-1, intercellular adhesion molecule; VCAM-1, vascular cell adhesion molecule. ^a^*p*<0.05.

The mediating effects of biochemical metabolic factors on the association between BMI and unfavorable inflammatory status and endothelial dysfunction are shown in Fig 1. The direct effect of BMI on an elevated hs-CRP level was significant (*ß =* 0.29, *p* < 0.001), while the indirect effects of biochemical factors were not significant (Fig 1A) after adjustment for four biochemical factors. Regarding the association between BMI and an elevated ICAM-1 level, the HDL-c level showed an independent indirect effect (BMI → HDL-c, *ß* = −1.09, *p* < 0.001; HDL-c → elevated ICAM-1, *ß* = −0.06, *p* = 0.03). However, the direct effect of BMI on an elevated ICAM-1 level was not significant (Fig 1B). Neither the direct nor the indirect effect of BMI on elevated VCAM-1 levels was significant (Fig 1C).

## Discussion

In this study, poor metabolic health, as assessed by cMets scores, was independently associated with hs-CRP and ICAM-1 levels in adolescents. Among the metabolic components, the risk of an elevated hs-CRP level increased with increasing BMI and the risk of an elevated ICAM-1 level increased with decreasing HDL-c. Moreover, a high BMI was directly related to elevated hs-CRP levels and indirectly related to elevated ICAM-1 levels via HDL-c.

Studies have shown that ICAM-1 and VCAM-1 levels are elevated in patients with atherosclerosis, and the baseline ICAM-1 level is reportedly significantly associated with incident myocardial infarction [21] and carotid atherosclerosis [22]. ICAM-1 and VCAM-1 have been proposed as early biomarkers of changes in the artery wall [23] and to play a major role in the development of atherosclerosis; therefore, both are considered preclinical markers of endothelial dysfunction. In addition, both ICAM-1 and VCAM-1 belong to the immunoglobulin superfamily, and so are markers of inflammation and are correlated with other inflammatory markers. Briefly, as atherosclerosis progresses, increased secretion of tumor necrosis factor (TNF)-α and IL-1 induces production of IL-6 in activated monocytes and vascular smooth muscle cells, which enhances hepatic CRP synthesis. In response to TNF-α and IL-1, macrophages and endothelial cells produce ICAM-1 and VCAM-1 [24–26]. Endothelial dysfunction can be caused by metabolic syndrome, smoking, and insufficient physical activity [27], and lesions in the vessel wall can begin to grow slowly during childhood and develop in adolescence and adulthood [25].

Similar to our study, a Young Heart Project 2000 study assessed the relationships among hs-CRP, ICAM-1, VCAM-1, and cMets in adolescents [6]; the main findings were consistent with our results. However, this study involved 1:1-matched overweight and normal-weight adolescents, so its summary statistics may differ from the general population of adolescents. Our study also found that cMets scores were independently associated with levels of ICAM-1 but not of VCAM-1. This is consistent with the results of a study involving adults [11]. Regarding the association between metabolic components and markers of endothelial dysfunction, a positive association with TG [28] and insulin resistance [29], and a negative association with HDL-c [30] have been reported. We found a significant relationship between HDL-c and ICAM-1. This may be because HDL-c inhibits expression of endothelial adhesion molecules [31]. By contrast, there was no significant association between metabolic health and VCAM-1 levels. The biological responses of ICAM-1 and VCAM-1 have been reported [25,32], but the mechanisms underlying the changes in ICAM-1 and VCAM-1 levels are unclear [6,33]. In addition, it has been proposed that ICAM-1 may be predictive of cardiovascular events in adults, and VCAM-1 may be a prognostic factor in patients with atherosclerosis [25]. Therefore, caution is needed when interpreting the clinical implications of VCAM-1 levels in children and adolescents.

hs-CRP is a nonspecific marker of inflammation and evidence has implied that hs-CRP levels are associated with metabolic syndrome and are predictive of CVD development [10, 11]. Although the relationship between metabolic components and hs-CRP levels is unclear, several studies in children and adolescents [6,9,16], and in adults [10,11] have reported a significant positive association between metabolic syndrome and hs-CRP levels. In addition, hs-CRP levels are reportedly correlated with the severity of CVD. A case study in patients with angina reported that the number of metabolic syndrome components was positively associated with the severity of coronary atherosclerosis and hs-CRP levels were positively correlated with the number of metabolic syndrome components and the severity of coronary atherosclerosis [34]. A previous study also reported that BMI was independently associated with elevated CRP levels [9], in agreement with our results. Furthermore, the 2-year Preventing Overweight Using Novel Dietary Strategies (POUNDS) LOST study revealed that weight loss exerted a favorable effect on hs-CRP levels [35]. A longitudinal follow-up study also found that subjects with rapid weight gain had a higher risk of elevated CRP [36]. In this study, BMI, but not the other metabolic components, was directly associated with elevated hs-CRP levels. The association of BMI with elevated ICAM-1 levels was mediated by HDL-c. Therefore, BMI was directly or indirectly related to high hs-CRP and ICAM-1 levels, both markers of CVD. BMI was also associated with unfavorable metabolic conditions (high MAP and TG and low HDL-c). A possible mechanism underlying these associations may be that the composition and function of adipose tissue are altered in obesity, leading to the synthesis and secretion of various inflammatory mediators (e.g., adipokines, cytokines, and chemokines). Adipokines produced in adipose tissue help to regulate lipid metabolism, immunity, and inflammation processes, and are also associated with subsequent endothelial dysfunction [37]. Our results support this mechanism, and it is noteworthy that the relevance was confirmed in an observational study of adolescents rather than an experimental study. Childhood obesity can lead to obesity in adulthood [38]. Therefore, to reduce the risk of CVD, it is important that normal weight be maintained from a young age.

There is need for caution in interpreting the findings of this study. Because the subjects were not representative of the South Korean population, the generalizability of the results is limited. However, this study involved the general population rather than being a case-control design. Also, this was a cross-sectional study, so the temporal relevance is unclear. Errors in biomarker measurements can lead to non-differential bias, rendering the estimated values meaningless. We have not evaluated various indicators related to inflammation or endothelial dysfunction, such as TNF-a and IL-6. Therefore, further large-scale studies are needed to evaluate the feasibility of the study results for various indicators. Nevertheless, our findings have shown significant relevance that is consistent with previous studies. In addition, because various factors contribute to the development of CVD, we considered a number of covariates. However, the residual effects of unmeasured factors may have affected the results. Finally, the direct and indirect effects of BMI on an unfavorable inflammatory status and endothelial dysfunction were evaluated by mediation analysis, which may help further understanding.

In summary, poor metabolic health, as assessed by cMets scores, was related to an unfavorable inflammatory status and endothelial dysfunction in adolescents. Hence, maintaining metabolic health, particularly maintaining a normal weight, may prevent the development of CVD in later life.
